# Insect Pests and Arthropods in Heritage Interiors

**DOI:** 10.3390/insects17030309

**Published:** 2026-03-12

**Authors:** Peter Brimblecombe, Pascal Querner

**Affiliations:** 1 Department of Marine Environment and Engineering, National Sun Yat-sen University, Kaohsiung 80424, Taiwan; 2 School of Environmental Sciences, University of East Anglia, Norwich NR4 7TJ, UK; 3 Natural History Museum Vienna, Burgring 7, 1010 Vienna, Austria; pascal.querner@boku.ac.at; 4 Institute of Conservation, University of Applied Arts Vienna, Salzgries 14/3, Stock, 1010 Vienna, Austria; 5 Department of Integrative Biology and Biodiversity Research, Institute of Zoology, University of Natural Resources and Life Sciences, Gregor-Mendel-Straße 33, 1180 Vienna, Austria

**Keywords:** Austria, island biogeography, ecological community theory, insect traps, catch rate, libraries, galleries, museum stores, heritage ecosystem

## Abstract

The insect threat to heritage objects can increase with climate change, increased travel, movement of goods and loan exhibitions. This study used catch from 30 heritage environments across Austria. Overall arthropod catch rate in storerooms was lower than in museums and libraries. Taxonomic richness of the ecosystem in the buildings was a product of building size, perhaps paralleling island biogeography. Heritage pests are distributed independently and follow environmental gradients, perhaps aligning with Henry Gleason’s continuum theory of ecological communities. Catch rates for some abundant pests are evenly distributed among buildings (e.g., *Psocoptera* booklice, *Lepisma saccharinum* common silverfish), but *Tineola bisselliella*, the webbing clothes moth, is unevenly distributed because some locations have large infestations. Rare species are unevenly distributed, as these are found in only a few buildings. A characteristic set of insect pests appear to dominate indoor heritage environments in Austria: *Psocoptera*, *Lepismatidae* silverfish, *Tineola bisselliella* webbing clothes moth and carpet beetles like *Anthrenus* spp. and *Attagenus* spp. These pests are also common in the interiors of heritage buildings in some other European countries.

## Simple Summary

Museums, galleries, libraries and storage facilities are at risk from damage to their collections by insects. This risk can increase under a changing climate or with the transport of invasive pests imported with exhibition loans and other materials or via more frequent international travel. We studied the insect pest and arthropod community in 30 heritage buildings in Austria over multiple years. The number of insects trapped in storerooms was low compared to museums and libraries. Museums in urban areas appear to resemble isolated islands, so transfer of individuals, species or populations between them is limited. The number of different species found in the museum ecosystem appears to be associated with the size and complexity of the building and its collection. Individual insect species are distributed independently of each other and do not compete for food, water or habitat. This could be because the museum pest community is of low diversity and has a simplified food web based on detritus. This leads to populations being controlled by environment and climate factors. Our study helps to understand and manage pests in ways that avoid the use of harmful pesticides.

## 1. Introduction

Insects have been pests within buildings since pre-history. They have spread disease [1], damaged crops [2], consumed stored food [3] and damaged materials [4] and are part of the urban ecosystem [5]. Termites and wood-boring beetles can cause extensive damage to furniture and building fabric [6,7,8]. These pests are a continuing threat to objects on display in museums, galleries and libraries [9,10,11,12]. They damage organic objects by chewing or tunnelling, leaving behind holes, frass (insect excrement) and webbing. Termites and wood-boring beetles can damage wooden objects, sculpture and furniture [7]. The larval stage of some insects feed on the starch-based adhesives in older book bindings and may leave tracks through the pages of books. Paper and document destruction arise mainly from silverfish, beetles, termites and booklice. Textile damage is often caused by clothes moths and carpet beetles, which can infest historic fabrics, carpets, upholstery and clothing, causing irreversible damage. In addition to this direct damage, the presence of insects can harm the reputation of heritage properties and may suggest a lack of care for the collection [13].

The threat continues to be serious because of changes in insect populations (i) as a function of warming conditions [14,15], (ii) due to increased travel and the movement of goods and (iii) with the rise of pests traveling with loan exhibitions [16,17]. Furthermore, concern over the environmental and health problems that result from the use of persistent pesticides has meant increasing pressure to restrict their use [18]. Adoption of a sustainable approach to the control of museum pests using an integrated pest management (IPM) strategy has become more common across heritage institutions, which means they frequently monitor insects [18,19] and limit the application of biocides. Such approaches may benefit from viewing pests in the heritage environment more as a community within a restricted ecosystem and completely artificial environment. Heritage interiors are artificial habitats, compared to natural ecosystems such as forests, grasslands, lakes, rivers or reefs, even though these are under anthropogenic influence. Urban ecosystem studies usually focus on outdoor habitats of parks, forests or green roofs, but indoor habitats are rarely studied over longer periods to show changes in the arthropod or insect pest communities.

The availability of food may be one additional constraint; nevertheless, heritage collections can provide large quantities of skin, fur, paper and other organic material along with fragments of food from catering. Although it is likely that the heritage environment has relatively few trophic levels, there may be simple food webs within the buildings. Some insects take advantage of secondary resources, such as dead insects and organic detritus [20]. While ecosystems typically have some kind of stability, there can also be striking variations. For example, resource pulses may lead to a rapid change in insect abundance following events such as a food spill or water leak, and fungal growth can trigger sudden expansion, appearing as an infestation. This can affect the population dynamics of colonization, but additionally, periods of low visitor activity may also enhance insect abundance, e.g., closures during COVID-19 [21]. Reproduction and life cycles could benefit from the warmer temperatures of the future, and more constant indoor temperatures allow for year-round breeding, unlike seasonal constraints outdoors [14]. It is also important to consider potential competition for food or suitable niches. Additionally, predation and parasitism play a role: spiders, centipedes or parasitoid wasps can act as natural control agents [22]. Museums and building indoor environments in general lack extensive photosynthesis, so a base for a productive ecosystem (i.e., plants, bacteria, algae and archaea) is missing, so there are no new organic materials from photosynthesis. This limits insect activity and is the reason that most museums do not allow flowering plants in galleries and storerooms.

Besides insects and arthropods permanently living indoors, other species enter from the external environment via doors, windows, vents, etc. The animal communities found inside buildings are a mixture of both groups. The taxa reproducing inside and that also utilize food within the building (mostly as larvae) make up most of the animals found. However, in unsealed buildings there can be large numbers of accidental invaders. This is also dependent on the building surroundings, such as concrete “deserts” in cities, as compared with parks in urban locations or agricultural and natural habitats in rural areas. As an ecosystem, the heritage environment is likely to resemble islands and lacks connectivity along ecological corridors. Some buildings will be separated by agricultural or natural rural spaces, while others will be more urban [23]. Transport between heritage locations may have few corridors along which to move, perhaps carried in packaging from exchanged collections [16] and plants. It seems that “museum ecology”, like “island ecology”, would be shaped by isolation, habitat size, immigration and extinction, along with clear boundaries to the outdoors, all influencing its local diversity. This could produce distinctive patterns of biodiversity, evolution and ecosystem function. Museums have a limited amount of biological activity and rather few habitat types (gaps along the walls, dead spaces under floorboards, attics, basements, etc.) [24]. There is a reduced species richness compared with typical terrestrial ecosystems, and richness is a balance between long-term presence of some species, immigration flow and extinction rates.

We are particularly interested in the “ecology of the heritage environment” with a focus on those pests damaging material in collections. This paper attempts to structure the catch of insects present in Austrian heritage museums, libraries and galleries within the framework of an ecological community. We have been interested in the habitats provided by museums, such as attics and basements or microhabitat cracks, air gaps, and insulation spaces, along with display furniture. It is of special importance to consider places such as storerooms that are rarely disturbed by human activity [25,26]. This study will explore whether there is a particularly characteristic insect community or biome [19]. Does the community structure align with the theory of individualism imagined by Henry Gleason [27]? Within this concept, species change independently in relation to other species present. They respond independently to environmental gradients rather than forming tightly integrated, interdependent units. This seems likely to be a feature of heritage interiors, as museums, libraries and storage facilities are rather barren environments, with biota limited in trophic levels, containing fragmented populations typically of low density.

Despite a long acquaintance with the problem and numerous surveys of museum pests, there have been few systematic studies that encompass larger numbers of institutions. An early survey of natural history museums attempted to give a sense of the pests that need to be controlled [28]. English Heritage maintains a large database of trapped insects from their properties [29]. In more recent years the *WhatsEatingYourCollection* (WEYC) survey in the UK was assessed [30]. Conferences on IPM have presented data, but surveys across multiple institutions remain rare.

We also want to understand if specific insect communities are associated with different types of collections or room use, e.g., libraries, galleries, museums and storerooms, and to additionally make some comparisons with the museum catch in other parts of Europe. The notion of a community within the indoor environment can have practical implications for IPM because of the risks posed by heritage insects. It may also be useful to view indoor spaces ecologically, as it shifts the focus from simply eradicating the pests to managing habitats and their ecology. This information can help to reduce or eliminate insect populations by controlling indoor environmental conditions that are favorable for their development.

## 2. Materials and Methods

### 2.1. Study Locations

**Figure 1:**
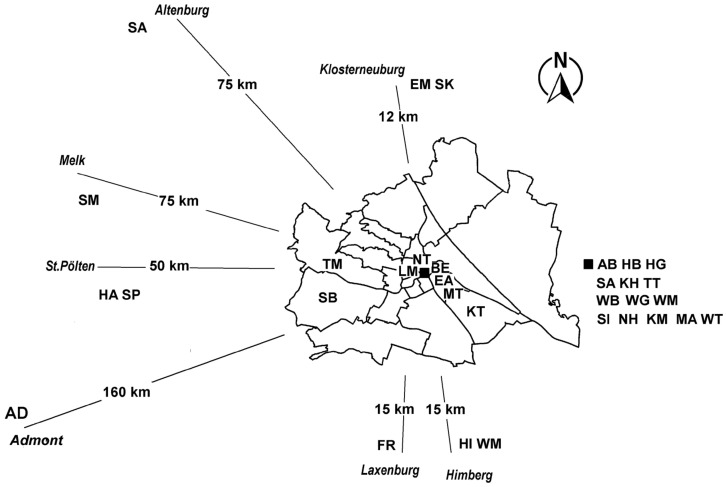
Building locations in the districts of Vienna (outlined) and eastern Austria. The locations in eastern Austria are not to scale but show the direction from the center of Vienna along with the distances from Innere Stadt (the first and central district of Vienna), marked by a black square. The two-letter location codes are listed in Table 1. Locations that are in Innere Stadt are marked next to the black square to the right of the Vienna region.

This research was conducted at 30 study locations (i.e., buildings) in Austria, including museums, as shown in the schematic map of Figure 1. It gives a sense of direction to the sites from central Vienna (Innere Stadt). The locations cover a range of urban environments through to rural locations, with some based in small towns. These locations were chosen to cover a range of building types: (i) modern storage with good climate control, with interiors well-sealed from the outdoor environment, (ii) historic buildings with heating, (iii) historic buildings with heating, ventilation and air conditioning and (iv) historic buildings without any active climate control. The libraries were typically historic buildings, often in large monasteries. Museums varied from large national institutions in grand historic buildings (the Natural History Museum or Oberes Belvedere, for example) with large numbers of domestic and international visitors to smaller specialist collections in less substantial buildings. The storage facilities were generally not open to the public and therefore much less visited. Table 1 lists the study locations along with details of the insect trapping program in each. We were not able to use the real names for all institutions as some preferred to remain anonymous.

### 2.2. Traps and Insect Identification

The trapping program used sticky blunder traps (Catchmaster, Bayonne, NJ, USA) of cardboard with pressure-sensitive glue that were typically placed at regular intervals (every 10 m or so) along the walls or under windows or next to doors and other entrances. A few pheromone traps [31] for the webbing clothes moth (Finicon, Insektenvernichter, Munich, Germany) were also added to monitor the activity of this common museum pest, although they also trapped other insects. In most cases, one of these traps was set out on the floor in each room under study. The contents of the traps were examined and species identified three to five times per year. Traps were replaced when full or in spring and autumn of every year.

Identification of the arthropods caught on sticky traps utilized different keys [32,33,34] and was based on morphology. We followed the increasingly recommended practice of writing all taxonomic levels in italic script [35]. As insects that harm objects in collections are the main concern in museums, more of our attention focused on pests, and these were identified to the species level where possible. Other taxa, such as spiders, true bugs, centipedes or millipedes, were also counted but not identified to the species level.

### 2.3. Data Recording and Analysis

The individual arthropod species numbers were recorded on spreadsheets, although at times these were recorded as genera or higher taxonomic ranks for some arthropods. It was necessary to compare the catch between different buildings, which often involved different numbers of traps set for different periods of time. This meant correcting for differences in trapping effort. In this study, the effort was typically expressed as the annual catch rate *R*_B_ = *C*_B_/(*N*_B_ *t*_ex_), which for a building, B, is the number of insects caught in a year (*C*_B_) divided by the number of traps (*N*_B_) and the years of exposure (*t*_ex_). Variation in this effort, when plotting species saturation curves [36], was expressed by sampling different numbers of trapping sites selected randomly within the buildings.

Gini coefficients have been used here to represent skewed catch rate data; although traditionally used in economics, they have proven to be effective in biology [37]. Although the Shannon index is often used in pest ecology to account for species biodiversity [38], we adopted the Gini coefficient here because the Shannon index requires logarithms, so a zero catch presents a mathematical difficulty. Here, the Gini coefficient was used to describe the distribution of insects among the sites. Species saturation curves were modified in this study to represent the cumulative number of taxa observed at the locations as a function of the cumulative effort.

Given the integer nature of the trapping data and the likelihood of skewed distribution, because many traps had no insects, we used non-parametric statistics and reported median and lower (*Q*_1_) and upper quartiles (*Q*_3_). Box-and-whisker plots rather than mean and standard deviation were used to display, for example, the differences in catch between buildings. Kendall’s *τ* was taken as an effective measure of the association between two ranked variables [39], as it is less sensitive to outliers than Spearman’s *ϱ* or Pearson’s correlation *r*. The statistic *τ* was used when comparing the relationship between the catch and the number of traps or comparing the catch rate of different insect pests. Kendall’s test is generally robust when dealing with small sample sizes, and *τ* resembles the familiar parametric correlation coefficient *r*. As mentioned in the results, the Mann–Whitney and Kruskal–Wallis tests were used to determine the significance of differences between data sets, especially when examining catch rates between different building types.

## 3. Results and Discussion

### 3.1. Catch and Catch Rate

**Figure 2:**
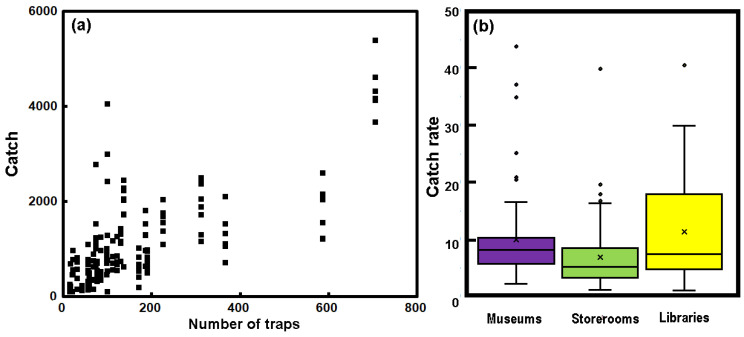
(**a**) The total annual catch as a function of the number of traps set out in the buildings. (**b**) The annual catch rate for the different classes or heritage interiors. The boxes represent the lower and upper quartiles, with the median denoted by the line across the box, with a cross to represent the mean. The whiskers show the range of all other points, except those that are deemed as outliers, i.e., values that lie over 1.5 times the IQR beyond the box.

The total annual catch for arthropods from all the buildings studied here is shown in Figure 2a as a function of the number of traps. The numbers of traps are constant from year to year, so the points often appear as vertical sets, which give a sense of the interannual variation in catch. The Kendall test suggests a significant degree of correlation between catch and the number of traps (*τ* = 0.53; *p*_2_ < 0.0001). Because the locations differ so much in terms of the trapping activity (i.e., number of traps set out), any comparison needs to account for the number of traps. Thus, it is often necessary to think in terms of the number of insects caught for a given amount of trapping effort. This was expressed as the annual catch.

The catch rate is displayed for the three different types of building interiors, museums, storage areas and historic libraries (Figure 2b). Although the catch rate shows considerable variation, as illustrated by the box-and-whisker plot, there is a statistically significant difference between the catch rates determined in the different building types (Kruskal–Wallis *H* = 19.1; *p* < 0.0001). The catch rate in storerooms is lower than that of museums and libraries. The median catch rate in museums at 8.23 is significantly higher than in libraries, with a median of 6.1 (Mann–Whitney *p*_2_~0.02) and of 5.45 in storerooms (Mann–Whitney *p*_2_ < 0.0001).

### 3.2. Taxa

#### 3.2.1. Taxonomic Richness

**Figure 3:**
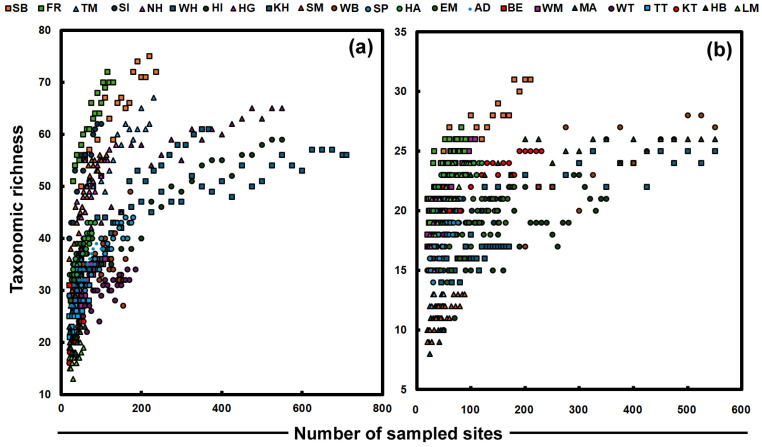
Taxonomic saturation curves assembled from a varying number of trapping sites sampled randomly from each location for (**a**) arthropods and (**b**) indoor arthropods.

More than a hundred different arthropod taxa were distinguished in the traps. These traps caught more than 200,000 individuals. We have termed the number of taxa at given sites as taxonomic richness. This is loosely related to species richness, but in our work, it is not identical, as not all arthropods could be determined to the species level. The total species richness is much higher than the taxonomic richness, as all spiders, for example, were classified as spiders but probably belonged to ten or more species. Figure 3 presents taxonomic saturation curves for arthropods. It was determined by taking random subsets of the trapping sites from each building. The richer locations (such as FR and SB) seem to level out at about 55–65 species when larger numbers of sites are chosen. Figure 3a shows that at these locations, more species might be expected, but when we look at many of the buildings, the number of taxa does not increase, such that only a few new species are found with increased resampling, i.e., the trend becomes almost level. The locations that show the steepest curves are all surrounded by large parks (SB, FR and TM) and have a high diversity, mainly because many species are more typical of the outdoor environment. However, these species cannot survive long indoors; they do not reproduce inside and cause little damage to collections, although they may represent food for the larvae of *Anthrenus* spp. and *Attagenus* spp.

If museum buildings are surrounded by natural habitats, they enhance indoor diversity through increasing the total number of species. Thus, although museums often resemble isolated islands, they can be influenced by directly bordering habitats, such as parks or forests, which are ecologically richer environments in terms of arthropods. In contrast, locations surrounded by city streets, concrete and other buildings are likely to contribute fewer invading species to heritage interiors. Most of the arthropods originating from natural habitats are only accidental visitors that enter the building by chance, not as an active invasion. These arthropods search for food, hide in gaps in the buildings, or are attracted by the different levels of light or warmth within, but they are not well adapted to the indoor environment (e.g., too dry, with limited food).

Figure 3b shows the taxonomic richness of indoor-dwelling arthropods (Appendix A) as a function of the number of sites considered. This data is rather scattered compared with that of Figure 3a because of the more limited number of species collected. Of the arthropods that live entirely indoors, only 46 different types were found in our work. Schönbrunn palace (SB) has the steepest curve with the highest number of species. The palace is a very large historic building in a park with many visitors and numerous niches associated with different floors and rooms. Transport of new indoor species to heritage interiors is probably related to the import of materials indoors, especially packaging [16]. Silverfish, such as *Ctenolepisma longicaudatum* or *C. calvum*, do not survive outside, so they have to be transported into the building, perhaps on exhibition materials. Species richness [40] can be used to guide resource management [41]. Given that museums might be seen as islands in a sea of urban structures, it may be possible to consider richness in relation to island biogeography [42]. On islands, comparative species richness may be related to the size [43,44,45], area, length of coastline or edge, or habitat diversity of the island or remoteness from coasts or the mainland [46]. Recent reviews suggest a relationship between species richness and productivity, such that productivity affects species richness at large scales, but there is uncertainty regarding the underlying mechanisms [47]. In the case of the heritage locations studied here, size (perhaps as the total area of the building or wall perimeter) or habitat diversity is difficult to ascertain in a quantitative way. It might be related to the room area [48], building footprint, visitor numbers, size of the collection or amount of material exchanged. The locations studied here all revealed the presence of numerous taxa. Even at locations with few traps (BA, AD, LM and EA), richness values of 10, 20, 21 and 28 are found. In the buildings studied, the slopes of the taxonomic richness against the number of sites were of a similar slope, especially where the number of sites sampled is small.

#### 3.2.2. Relative Catch

**Figure 4:**
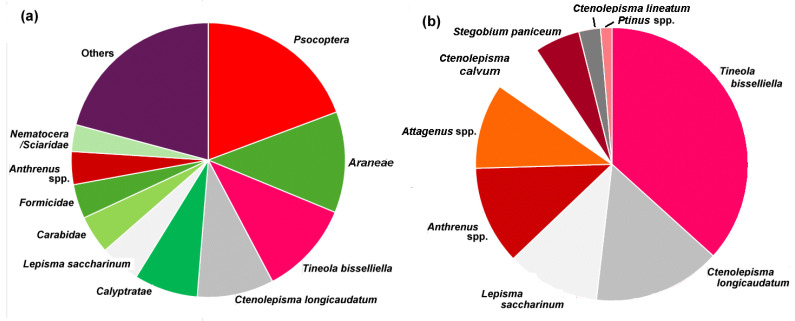
(**a**) The catch of the main types of arthropods trapped in the buildings. Insect pests are in red–pink, with silverfish in grey. Arthropods that are not pests are in green, with other catches in lesser amounts in purple. (**b**) The average catch as a proportion for each site for the dominant insect pests from the heritage interiors.

From the more than 200,000 individuals caught, the catch of the ten most common arthropods from the traps (i.e., more than 7000 individuals caught for each species) are shown in Figure 4a. “Others” encompass different taxa with some 43,000 individuals trapped in lesser quantities. *Psocotera* or booklice dominated the catch (~30,000 individuals), followed by *Aranaea* (spiders) and then *Tineola bisselliella* moths (~23,000 individuals). However, silverfish (*Lepismatidae*), occurring as four different species, amounted to more than ~34,000 individuals. There were significant catches of *Calyptratae*/*diptera* (cluster flies), *Carabidae* (ground beetles), *Formicidae* (ants) and *Nematocera* and *Scuridae* (other flies).

Figure 4b shows the distribution of pest species in the catch but as proportions in each building so as to not be dominated by the buildings where very large numbers of traps were set out. Pests commonly found in Austria included *Psocoptera* (book lice); although a potential source of damage in libraries [49], they are not especially harmful in the Austrian institutions examined here. Moths (*Tineidae*), such as *Tineola bisselliella* Hummel, 1823 (webbing clothes moth) [50], were found in our study along with lesser numbers of *Tinea pellionella* Linnaeus, 1758 (case bearing clothes moth) [51]. which harms wood and fabrics.

Carpet beetles (*Dermestidae*) were found in many locations and included *A. verbasci* Linnaeus, 1767 (varied carpet beetle), *Anthrenus museorum* Linnaeus, 1761 [52], *A. olgae* Kalík, 1946, *A. scrophulariae* Linnaeus, 1758 (common carpet beetle), *A. fuscus* Olivier, 1789, *Attagenus smirnovi* Zhantiev, 1973 (brown carpet beetle or vodka beetle) [15], *A. unicolor* Brahm, 1971 (black carpet beetle) and *A. pellio* Linnaeus, 1758 (fur beetle). These genera were at times not recorded to the species level as the larvae can be difficult to distinguish.

Four *Lepismatidae* (silverfish) were found at the heritage locations studied here (Figure 4b): *Lepisma saccharinum* Linnaeus, 1758 (common silverfish), *Ctenolepisma longicaudatum* Escherich, 1905 (longtailed silverfish) [53,54], *C. lineatum* Fabricius, 1775 (four-lined silverfish) [55] and the ghost silverfish *C. calvum* Ritter, 1910 (or *C. phantasma*, see the description of the new species [56]). Whether all the ghost silverfish found in Austria or Central Europe [57] are *C. calvum* [56] or are the new species remains to be determined.

*Stegobium paniceum* Linnaeus, 1758 (biscuit beetle) [Ignatowicz] and *Ptinus* spp. Linnaeus, 1767 (spider beetles) [58] were relatively abundant (Figure 4b). Additionally, a range of beetles were found in lesser numbers as well as some species of woodboring beetles: *Anobium punctatum* De Geer, 1774 (furniture beetle) [59], *Priobium carpini* Herbst, 1793, *Hexarthrum exiguum* Boheman, 1838 (pit prop beetle), *Oligomerus ptilinoides* Wollaston, 1854 (southern furniture beetle) [60], *Hylotrupes bajulus* Linnaeus, 1758 (house longhorn beetle) [19] and *Lyctus sp.* Fabricius, 1792 (powder-post beetles) [61], *Thylodrias contractus* Motschulsky, 1839 (odd beetle) [62], *Reesa vespulae* (skin or American wasp beetle) and *Trogoderma glabrum* Herbst, 1783 (glabrous cabinet beetle) [63].

The catch of *Ptinus* spp. (as two different species), shown at “12 o’clock” in Figure 4b, at a total of 1331, is the smallest total from the 30 locations. After this genus, the numbers caught drop rapidly to the next most abundant, *Hexarthrum exiguum* (pit-prop beetle), with a catch from the locations of just over 400 (Table 2). Thus, *Ptinus* spp. represented a good cut-off for Figure 4b. Abundant pest species are also widely spread across the locations. *Tineola bisselliella* (webbing clothes moth) and *Lepisma saccharinum* (common silverfish), for example, were caught at 29 of the 30 locations, while *Psocoptera* were found at 28 locations. However, if we group all four species of *Lepismatidae* (silverfish), then this order of insects was caught at all 30 locations, as was the genus *Anthrenus*, with the genus *Attagenus* found at 29 locations. Other species shown in Figure 4b were found at 23–25 locations (Table 2), so still rather widespread. Of those pests not plotted on the pie diagram, *H. exiguum* was found at only two locations. Although not particularly abundant, *Reesa vespulae* (skin or American wasp beetle) was found at eight locations but was still not especially widespread and typically occurred in urban buildings [17].

Clearly the insect pests shown in Figure 4b are widespread and 23 of the 30 locations had all of these insects present, although *Psocoptera* are not treated here, as they have not been found in association with objects in museums, libraries or archives in Austria over the last 20 years. If we consider *Lepismatidae* as a group, then 19 locations were complete with all of the insect groups represented. This illustrates that a small number of species account for much of the entire catch at many heritage locations. These abundant insects can amount to more than 90% of the catch from some buildings (median 57.4%; *Q*_1_ 52.3%; *Q*_3_ 66.8), so they often dominate the catch of pests, but at a few locations, the amount may be less than 30%, and here, the catch was dominated by other taxa such as spiders, ants, flies and ladybirds (shown in Figure 4a).

#### 3.2.3. Interspecies Correlation

It seems possible that the catch of insectivores might be correlated with the trapped pests. Two predators present in the heritage environment were the order *Araneae* (spiders) and class *Chilopoda* (centipedes). *Araneae* feed on smaller insects of the genera *Anthrenus*, *Attagenus*, *Anobium*, the family *Lepismatidae* (silverfish) and flies of the suborder *Nematocera* and family *Sciaridae*. Anobiids were rather rare in the traps studied here, but there was a positive correlation between the catch of *Araneae* and that of *Lepismatidae*, *Anthrenus*, *Attagenus*, and *Nematocera/Sciaridae* (Table 3), although the correlation with *Attagenus* was weak (*p*_2_ = 0.136). These spiders catch insects from the floor and not in webs. The correlation of *Chilopoda* with the insects shown in Table 3 was only significant for *Lepismatidae*. This was a little surprising, as *Chilopoda* prey on flies, yet the correlation with *Nematocera/Sciaridae* was not strong. However, *Chilopoda* correlated rather well (Kendall’s *τ* = 0.267; *p*_2_ = < 0.04) with the abundant *Calyptratae* (cluster flies).

**Figure 5:**
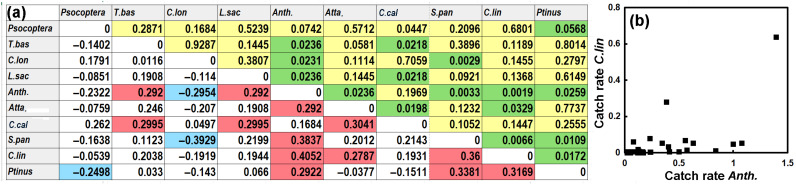
(**a**) Nonparametric Kendall’s correlation between annual catch rate of the ten common pest taxa at various locations. Upper-right diagonal gives the *p*-values (yellow and green), with those less than 0.05 shaded in green. The lower-left diagonal gives the value of Kendall’s τ, with those that were significant at *p* < 0.05 and with negative correlations shaded blue and those that were positive shaded in pink. (**b**) Catch rate of *Ctenolepisma lineatum* as a function of catch rate of *Anthrenus.* Note: T.bas, *Tineola bisselliella*; C.lon, *Ctenolepisma longicaudatum*; L.sac, *Lepisma saccharinum*; Anth., *Anthrenus* spp.; Atta., *Attagenus* spp.; C.cal, *Ctenolepisma calvum*; S.pan, *Stegobium paniceum* and C.lin, *Ctenolepisma lineatum*.

The insect pests trapped in the study might be negatively correlated with other pests if they were competing for food or habitats. However, the four most abundant types of insect pests, *Psocoptera*, *Tineola bisselliella*, *Ctenolepisma longicaudatum* and *Lepisma saccharinum*, showed no significant inter-correlation, with *p*-values typically >0.15 (yellow diagonal on the left in Figure 5a). Kendall’s *τ* values were low < |0.2| (top rows of lower diagonal). Kendall’s *τ* was used instead of the more common Pearson regression coefficient *r* because the data appeared to be non-normally distributed (i.e., it was usually positively skewed).

Figure 5a shows frequent occurrences of lower *p*-values, suggesting significant correlations. In particular, *Anthrenus* spp. is significantly correlated (*p* < 0.05) with *Psocoptera*, *C. longicaudatum*, *L. saccharinum*, *Attagenus* spp., *S. paniceum*, *C. lineatum* and two species of *Ptinus* spp., as shown in pink. The correlation of *Psocoptera* and *Ptinus* spp. is negative, as are the correlations of *C. longicaudatum* with both *Psocoptera* and *Anthrenus* spp. (shown in blue in Figure 5a).

While the statistical parameters may be satisfying, the visual association in *x*-*y* plots of *C. lineatum* with *Anthrenus* spp. is not particularly convincing (Figure 5b). The scattered plot of the relationship with taxa suggests that the correlations found are relatively weak. However, they are mostly positive, which suggests that museums with conditions favorable to pest insects tend to be favorable to many different taxa and that they are environments generally susceptible to insect pests. The lack of negative correlations suggests that there is little evidence of competition between pest insects. This lack of competition between pest insects and the generally weak correlations seem to hint at independence.

#### 3.2.4. Distribution of Catch Rate

**Figure 6:**
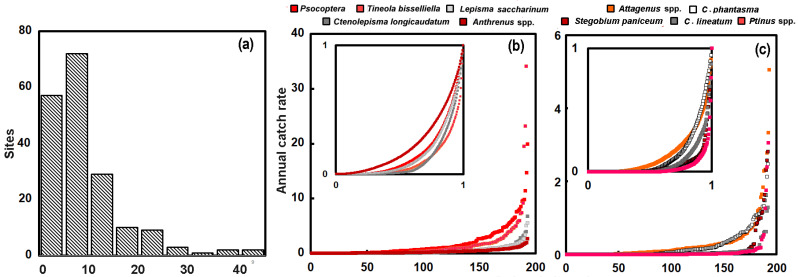
(**a**) Catch rates for all arthropods from all locations for each year of sampling. (**b**) Catch rates for all the *Psocoptera* and the four most common pests from all locations each year, arranged from lowest to highest. The inset shows a Lorenz plot of the catch rates. (**c**) Five less common, although characteristic, museum pests from all locations each year. The inset shows a Lorenz plot of the catch rates.

As illustrated in Figure 2, the catch of arthropods at the locations studied is variable. The distribution is skewed, as shown by the histogram in Figure 6a, which presents the catch rates for all arthropods from all locations for each of the years sampled. The annual catch rates for *Psocoptera* and the four most common pests *T. bisselliella*, *C. longicaudatum*, *L. saccharinum*, and *Anthrenus* spp. are plotted in Figure 6b as a Lorenz curve [64]. The catch rate from each year from each site is arranged from lowest to highest catch rates on the *x*-axis and the catch rate is plotted on the *y*-axis. This is shown in Figure 6b, with *Psocoptera* and *T. bisselliella* having the highest catch rates on the far-right-hand side. *Ctenolepisma longicaudatum*, *L. saccharinum*, and *Anthrenus* spp. have lower values for the maximum catch rates. It is easier to view the distribution if it is plotted with the values normalized to unity and shown in the inset. The shallowest curve (highest in the inset figure) is from *Anthrenus* spp., which suggests that this genus is more evenly distributed among the locations. If the catch rates were identical at all locations, the line would be a straight diagonal between zero and unity. The departure from an even distribution where all locations have the same catch rate can be expressed as a Gini coefficient (*G*), where zero represents total equality and unity represents absolute inequality. This is explained in the methods section, and values are listed in Table 2. The Gini coefficient for *Anthrenus* spp. is 0.5428, but for *T. bisselliella*, which is very unevenly distributed because it has very high catch rates at some locations, the Gini coefficient is larger at 0.7978. The two *Lepismatidae*, *C. longicaudatum* and *L. saccharinum*, have Gini coefficients of 0.772 and 0.705, while if all four *Lepismatidae*, were grouped together, the Gini coefficient would be 0.735. The Gini coefficient of *Psocoptera* is even lower at 0.675, suggesting a comparatively even distribution among the buildings.

Some abundant taxa (*Attagenus* spp., *C. calvum*, *S. paniceum*, *C. lineatum* and *Ptinus* spp.) with lower catch rates are plotted in the same way in Figure 6c. *Attagenus* spp. has the highest maximum catch rates at the locations but also the shallowest Lorenz curve (*G* = 0.714). The other insect taxa have higher Gini coefficients, which can be seen via the plotted points being pushed much more into the lower-right-hand corner of the inset compared to the inset of Figure 6b. *Pitinus* spp. is the most unevenly distributed, with a very high Gini coefficient of 0.925. *Reesa vespula* has an even higher Gini coefficient of *G* = 0.937, but it is present at just 13 locations, while *H. exiguum*, present at just two locations, has the highest coefficient, *G* = 0.974. An absence in the catch from the locations makes the distributions uneven.

It is possible to place the Austrian records gathered in the current study in an international context, as there are published lists of heritage pests from other countries. An early example is the survey of pests in natural history museums in the British Isles from the 1980s [28]. It focused on damage to collections rather than the catch and suggested the relative frequency of broad groups as follows: *Dermestidae* 47%, *Tineidae/Oecophorinae* (moths) 43%, *Ptinidae* 40%, *Psocoptera* 28%, *Lepismatidae* 25%, *Acariformes/Parasitiformes* (mites) 23% and *Blattodea* (cockroaches) 7%. These groups of insects roughly aligned with the catch found in our study.

The most common insects listed in the WEYC [30] database for 2010–2022 are *Anthrenus verbasci* (2186 records), *Liposcelis bostrychophila* (booklice; 2048 records), *Lepisma saccharinum* (2011 records), *Tineola bisselliella* (1553 records), *Hofmannophila pseudospretella* (839 records), *Tinea pellionella* (773 records), *Attagenus pellio* (720 records) and *Anobium punctatum* (568 records). There are naturally many caveats associated with this data, as discussed in the Supplement to WEYC [30], but it gives a rapid overview of the most frequently recorded insects in the UK heritage environment. Many of the records come from the English Heritage properties database, and the catch from these locations was discussed in an earlier paper [26]. This work explored catch statistics from ~30,000 traps set out between 1998 and 2012 and showed that the most frequent insect pests captured in English Heritage buildings were *Liposcelis bostrychophila* Badonnel 1931 (booklice; 65,622), *Lepisma saccharinum* (23,548), *Lathridiidae* Erichson 1842, i.e., the plaster beetle (6351), *Anthrenus* spp. (5701), especially as the varied carpet beetle, *A. verbasci* (1137) and *T. bisselliella* (882). In our study *C. longicaudatum* was found in much higher numbers than *L. saccharinum*, but fewer plaster beetles were found, suggesting that our heritage institutions are dryer.

Manachini [65] lists some insect pests in Europe, though from an Italian perspective. She was mostly interested in coleopteran native and alien species that cause damage, so the list may not be especially broad and, for example, does not include *T. bisselliella*. More recently, Hasnaoui et al. [66] reviewed pests at cultural heritage sites with a focus on France, providing a list of species, which are categorized as follows: category 1: the most common and hazardous species, category 2: species of lesser significance for the heritage domain and category 3: rarer species that may be present in heritage sites.

It is important to be a little cautious with insect lists from reviews, as they may reflect perceptions rather than actual catch. However, even in studies where insects are caught, the reporting of species can still reflect the conservation needs of the location being studied. Nevertheless, as perceptions are based on the experience of those involved in the protection of our heritage, these are still of value.

**Figure 7:**
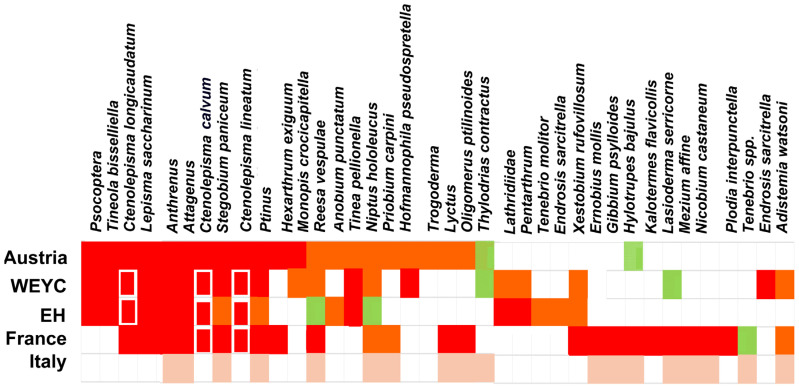
The relative data abundance of heritage insect pests from a number of sources. Note: color codes for Austria, WhatEatingYourCollection (WEYC) and English Heritage (EH) are as follows: red indicates > 50th percentile, orange indicates > 10th percentile, and green indicates present. In the French list, colors correspond to categories 1, 2 and 3, i.e., most to least important. Pale orange denotes presence on the list from Italy. The red rectangles for *Lepismatidae* denote the abundance after grouping all the silverfish.

The pest taxa are arranged in the order of the catch numbers from the Austrian sites in this study. The general picture that emerges from these reviews is shown in Figure 7. Here, the colors red–orange–green represent the highest to the lowest catches, as defined in the figure caption.

Figure 7 shows that although both *Psocoptera* and *T. bisselliella* are among the dominant pests in the catch from the Austrian and British datasets, they are not present in the review lists from France or Italy. However, both insects are certainly present in these countries [67]. In Austria, although *Psocoptera* are abundant, they are not observed to cause damage. In the case of *T. bisselliella*, it is hard to believe that the clothes moth is not a concern, as there is certainly concern over the problem it causes in stored products in France [68]. While *L. saccharinum* and *C. longicaudatum* are widely known, the other two species *C. calvum* and *C. lineatum* are more often noticed as pests in Central Europe [23]. In France, a number of species are found, including *Xestobium rufovillosum*, *Ernobius mollis*, *Gibbium psylloides*, *Kalotermes flavicollis*, *Lasioderma serricorne*, *Mezium affine*, *Nicobium castaneum* and *Plodia interpunctella*, but these are not noted as pests in the Austrian data. This should not be taken to mean that they are entirely absent in Austria, rather that they are not recorded as serious museum pests (e.g., *P. interpunctella* and *Gibbium psylloides*). *Kalotermes flavicollis* is a subterranean termite species that is only found in the south of France, Spain and Italy, and it is not known to occur in Austria or Germany.

### 3.3. Ecological Characteristics

The trapping programs revealed the types of arthropods present in heritage environments. Although the correlation between catch rates of different species in these buildings was not especially significant, the insect populations show some interesting patterns and characteristics.

In ecology, ecosystems and communities describe related but distinct levels of biological organization. Ecosystems emphasize flows of energy and cycling of matter and include abiotic entities. Given the lack of primary productivity in indoor environments, energy sources would typically rely on imported food. Community describes the populations living and interacting in the same area, the species present and their composition and interactions

It is possible to think about arthropods in heritage environments as if they are an ecological community. These heritage pests need to be tolerant of starvation and typically develop slowly, over months or years, and they need to be able to pause development (diapause) under unfavorable conditions. Heritage insects typically seem well adapted, e.g., *Anthrenus* larvae can survive years with minimal or no food, while the larvae of *T. bisselliella* can thrive on low-nutrient blends of wool [69]. In some cases, there may be predation within the broader indoor community, especially among *Araneae* (spiders) and *Chilopoda* but also *Malachidae* beetles.

A number of theoretical approaches have been developed to interpret community structures. The holistic theory sees a community defined by the interactions between the organisms in it, but this may not characterize museum pest populations given the low population density, reduced number of trophic levels, few habitat types and limited amount of biological activity.

However, the individualistic theory of Henry Gleason sees abundance in the species within a population that changes independently in relation to other species present. This theory sees individual populations changing along the environmental gradients. An independence among insect pests seems likely given the simplified food webs that are likely in heritage interiors. Here, the arthropod community is probably low-diversity, detritus-based and only weakly regulated by predators. The detritus in museums is typically organic materials in dust, skin fragments and dander, particles of human food and possibly fungi, though fungi are not generally active in the interiors studied here. The indoor populations seem to survive with relatively low numbers of reproducing individuals. In our cases, the indoor climate and the level of human disturbance such as visitor numbers or cleaning frequency are probably the most important ecological gradients.

In the buildings in our study, a higher sampling effort may not have the same effect as in natural habitats (e.g., grasslands or forests). Indoor habitats are very simple, and as there are no plants, there is no soil, and animals have few places to hide. In natural habitats, placing more traps will collect more species, as there are so many more niches. However, in buildings, there are a limited number of niches, so species richness curves can saturate relatively quickly. Indoor diversity can be low, such that, here, most sites seem only able to support the permanent presence of 25–35 arthropod species, even in larger buildings. This is a likely result of the simple food web, mostly supporting detritus feeders with a few predators taking advantage of the occasional open doors or windows to gain access to the interior.

Heritage pests are rather isolated in museums, libraries and storerooms, because insects need to be transported, sometimes over long distances (even internationally), into heritage buildings. This is a function of human activity, exhibition exchange, new object acquisition, etc. Transport mechanisms control the arrival of species (as with birds on islands), though in the case of museums, there is ingress through doors and windows and transport on materials or visitors. The frequency of colonization events for both islands and museums is likely an important driver of the populations to be found. The age of the heritage building and museum size are typically associated with a wider range of exhibitions, more visitors and a greater variety of indoor habitats, so larger museums might have more species, seemingly in line with the higher species richness of large islands [45,46]. Museum ecology, like island ecology, seems to be shaped by isolation, limited size and clear boundaries. This could produce distinctive patterns of biodiversity and ecosystem function. There is a reduced species richness compared with typical terrestrial ecosystems (soils of forest or grasslands, for example, have thousands of individuals and 50 or more species every 1 × 1 m^2^). Richness in these ecosystems is likely to be a balance between immigration rate and extinction rate.

## 4. Conclusions

The characteristic taxonomy in indoor heritage environments suggests typical species, such as *Psocoptera*, *Lepismatidae*, *T. bisselliella*, *Anthrenus* spp. and *Attagenus* spp. These are of concern, with moths, silverfish and carpet beetles likely representing a source of damage to collections.

The insectivores *Araneae* and *Chilopoda* were correlated with some pests that they prey on. Nevertheless, there was only a weak interspecies correlation among the pests. This suggests that the independent concept of community is likely to apply to the population found in heritage buildings, which is typically a nutrient-poor, low-density ecosystem with a small standing population. There are nevertheless occasional population explosions appearing as infestations. Widely separated habitats make it difficult for populations to transfer between museums.

There is much further work needed to understand heritage pests, and the large differences between activity and collections in buildings make them difficult to compare. In particular, it would be useful to understand what triggers pest infestations in heritage environments. Hopefully gaining more substantive evidence that ecological theories can be usefully applied to the heritage environment will give further guidance in the management of museum pests. Future work could examine the migration and introduction of new species into buildings. Our current studies look to climate as a limiting factor and should undertake a room-by-room analysis. It is important to better understand the gradients in climate, food quality and availability and habitat availability in museums. Additionally, we need to gain a better appreciation of the relevance of the metrics that effectively describe the catch rate in insects among rooms of various sizes, with different collections and visitor profiles. Analysis can be limited by small catch sizes, with traps often empty, which limits statistical certainty, so better statistical tools would be of value.

The broad analysis of insects in heritage buildings presented here nevertheless has some policy implications for the management of insect pests. Treating the pests as part of a community should allow us to recognize that the presence of some species might imply that others are likely to be found. It could also give a sense that there is a typical background community likely to be found in the heritage environment. The weak, though broadly positive, correlations among species suggest that some buildings have environments that are susceptible to insect pests, so these may need more active monitoring programs. Transport of pests with exhibition materials and new objects is an important way to transfer new species, emphasizing the relevance of careful inspection of incoming material. Seeing the heritage environment more as a community within a restricted ecosystem may ultimately benefit integrated pest management.

## Figures and Tables

**Table 1:** Locations that form part of the current study and the duration of monitoring in years. The code is two letters followed by a designator: libraries *l*, museums *m* and storeroom *s*. Climate control: heating ventilation and air conditioning (*HVAC*); *control*, heating and cooling throughout concrete core activation in the floor and ceiling, mobile de-humidifiers or humidifiers; *heating*, in winter season.

Code	Location	Setting	Climate Control	Duration /Years	Traps	Arthropod Catch
AB*s*	Albertina Storage	urban	*HVAC*	4	15	666
AD*l*	Admont Abbey	rural	*none*	7	100	12,094
BA*m*	Basteihalle	urban	*HVAC*	4	22	2736
BE*m*	Oberes Belvedere Museum	urban	*HVAC*	7	75	8917
EA*s*	Exilarte Zentrum Storage	urban	*HVAC*	5	17	1409
EM*s*	Klosterneuburg Storage	rural	*HVAC*	6	77	3115
FR*m*	Franzensburg Castle	rural	*none*	7	130	7871
HA*s*	Hart Storage	rural	*none*	7	85	4405
HB*s*	Archive and Studio	urban	*heating*	6	60	2081
HG*s*	Storage	rural	*heating*	8	137	15,053
HI*s*	Storage	rural	*control*	7	588	11,311
HM*m*	Heeresgeschichtliches Museum	urban	*heating*	8	47	5670
KB*l*	Capuchin Library	urban	*none*	3	21	648
KH*m*	Large Museum	urban	*heating*	6	710	26,573
KT*s*	Belvedere Storage	rural	*control*	4	56	916
LM*s*	Leopold Storage	urban	*HVAC*	7	56	4621
MAs	MAK Storage	urban	*none*	7	60	2005
NH*lms*	Natural History Museum	urban	*heating*	4	825	18,988
SA*l*	Altenburg Abbey Library	rural	*none*	7	32	4142
SB*ms*	Schoenbrunn Museum	urban	*none*	8	236	13,442
SI*m*	Sisi Museum	urban	*none*	7	100	5415
SK*l*	Klosterneuburg Library	rural	*none*	7	43	1197
SM*l*	Melk Library	rural	*none*	5	113	3930
SP*s*	Kulturdepot Storage	rural	*control*	7	191	5189
TM*ms*	Archive and Museum	urban	*heat*	7	245	17,537
TT*lms*	Specialist Museum	urban	*HVAC*	6	69	3074
WB*ls*	Storage	urban	*none*	7	172	4161
WH*s*	Storage	rural	*control*	6	367	7797
WM*m*	Museum	urban	*HVAC*	5	122	4028
WT*s*	Storage	urban	*heating*	6	186	7479

**Table 2:** Abundant insect pests indoors within the Austrian heritage environment. **Catch** represents the total catch of pests trapped from all the locations in this study, **Presence** is the number of locations where the pest was trapped (maximum 30) and **Gini** is the coefficient that represents the statistical dispersion, as described in the text.

Pest	Common Name	Abundance	Presence	Gini
*Psocoptera*	Book louse	41,591	29	0.675
*Tineola bisselliella*	Clothes moth	23,169	29	0.798
*C. longicaudatum* ^1^	Grey silverfish	16,826	25	0.772
*Lepisma saccharinum*	Common silverfish	10,681	29	0.705
*Anthrenus* spp.	Carpet beetles	7693	30	0.544
*Attagenus* spp.	Carpet beetles	6506	29	0.714
*C. calvum* ^1^	Ghost silverfish	5584	24	0.752
*Stegobium paniceum*	Biscuit beetle	2437	24	0.882
*C. lineatum* ^1^	Four-lined silverfish	1157	24	0.850
*Ptinus spp.*	Spider beetles	1331	23	0.925
*Hexarthrum exiguum*	Pit-prop beetle	432	2	0.974
*Reesa vespulae*	Skin beetle	196	13	0.937

^1^ *C* = genus *Ctenolepisma*.

**Table 3:** Nonparametric Kendall’s correlation between total catch rate of insectivores of the order *Araneae* (spiders) and class *Chilopoda* (centipedes) and some important taxa that are likely to be preyed on.

	*Lepismatidae*	*Anthrenus* spp.	*Attagenus* spp.	*Nematocera/Sciaridae*
***Araneae***				
Kendall’s *τ*	0.609	0.455	0.188	0.327
*p*_2_	<0.0001	<0.0003	0.136	0.009
***Chilopoda***				
Kendall’s *τ*	0.49	0.196	0.111	0.144
*p*_2_	<0.00015	0.128	0.395	0.265

## Data Availability

The insect trapping data used in this paper is available at: https://zenodo.org/records/18226647 (accessed on 3 March 2026).

## Appendix A. Indoor Arthropods

The following taxa were treated as able to survive and live permanently indoors.

*Anthrenus fuscus* (Olivier, 1789)
*Anthrenus museorum* (Linnaeus, 1761)
*Anthrenus olgae* (Kalik, 1946)
*Anthrenus querneri* (Holloway 2024)
*Anthrenus scrophulariae* (Linnaeus, 1758)
*Anthrenus verbasci* (Linnaeus, 1767)
*Argas reflexus* (Fabricius, 1794)
*Attagenus pellio* (Linnaeus, 1758)
*Attagenus smirnovi* (Zhantiev, 1973)
*Attagenus unicolor* (Brahm, 1790)
*Blattella germanica* (Linnaeus, 1767)
*Chelifer cancroides* (Linnaeus, 1758)
*Clogmia albipunctatus* (Williston, 1893)
*Corynetes coeruleus* (De Geer, 1775)
*Ctenolepisma calvum* (Ritter, 1910)
*Ctenolepisma lineata* (Fabricius, 1775)
*Ctenolepisma longicaudatum* (Escherich, 1905)
*Dermestes lardarius* (Linnaeus, 1758)
*Gibbium psylloides* (Czenpinski, 1778)
*Hexarthrum exiguum* (Boheman, 1838)
*Hofmannophila pseudospretella* (Stainton, 1849)
*Hylotrupes bajulus* (Linnaeus, 1758)
*Lepisma saccharinum* (Linnaeus, 1758)
*Monopis crocicapitella* (Clemens, 1859)
*Niptus hololeucus* (Faldermann, 1835)
*Oligomerus ptilinoides* (Wollaston, 1854)
*Oryzaephilus surinamensis* (Linnaeus, 1758)
*Plodia interpunctella* (Hubner, 1813)
*Priobium carpini* (Herbst, 1793)
*Ptinus fur* (Linnaeus, 1758)
*Ptinus sexpunctatus* (Panzer, 1789)
*Reesa vespulae* (Milliron, 1939)
*Scutigera coleoptrata* (Linnaeus, 1758)
*Sitophilus oryzae* (A.Hustache, 1930)
*Stegobium paniceum* (Linnaeus, 1758)
*Subfamily: Malachiinae* (Leach, 1817)
*Tenebrio molitor* (Linnaeus, 1758)
*Thylodrias contractus* (Motschulsky, 1839)
*Tinea pellionella* (Linnaeus, 1758)
*Tineola bisselliella* (Hummel, 1823)
*Tribolium castaneum* (Herbst, 1797)
*Trogoderma angustum* (Solier, 1849)
*Trogoderma glabrum* (Herbst, 1783)

*Genus Lyctus* (Fabricius, 1792)

*Family: Formicidae*
*Family: Bethylidae* (Forster, 1856)
*Family: Latridiidae* (Erichson, 1842)

*Order: Araneae* (Clerck, 1757)
*Order: Opiliones* (Sundevall, 1833)
*Order: Psocodea* (Hennig, 1966) often termed *Psocoptera*
*Order: Siphonaptera* (Latreille, 1825)

*Class: Chilopoda* (Latreille, 1817)
*Class: Collembola* (Lubbock, 1871)
